# From genes to patterns: five key dynamical systems concepts to decode developmental regulatory mechanisms

**DOI:** 10.1242/dev.204617

**Published:** 2025-08-01

**Authors:** Usha Kadiyala, David Sprinzak, Nicholas A. M. Monk, Shannon E. Taylor, Berta Verd, Katharina F. Sonnen, Lauren Moon, Adrienne H. K. Roeder, Ruben Perez-Carrasco, Pau Formosa-Jordan

**Affiliations:** ^1^ Department of Biophysics, University of Michigan, Ann Arbor, MI 48109, USA; ^2^ School of Neurobiology, Biochemistry, and Biophysics, George S. Wise Faculty of Life Science, Tel Aviv University, Tel Aviv 69978, Israel; ^3^ School of Mathematical and Physical Sciences, University of Sheffield, Sheffield S3 7RH, UK; ^4^ African Institute for Mathematical Sciences, Accra, GZ-192-2711, Ghana; ^5^ Biology Department, University of Oxford, Oxford OX1 3RB, UK; ^6^ Hubrecht Institute-KNAW (Royal Netherlands Academy of Arts and Sciences), University Medical Center Utrecht, 3584 GR Utrecht, The Netherlands; ^7^ Department of Physiology, Development and Neuroscience, University of Cambridge, 4, 7 Downing Pl, Cambridge CB2 3EL, UK; ^8^ Weill Institute for Cell and Molecular Biology and School of Integrative Plant Science, Section of Plant Biology, Cornell University, Ithaca, NY 14853, USA; ^9^ Polyploidy Integration and Innovation Institute; ^10^ Department of Life Sciences, Imperial College London, South Kensington Campus, London SW7 2AZ, UK; ^11^ Department of Plant Developmental Biology, Max Planck Institute for Plant Breeding Research, 50829 Cologne, Germany; ^12^ Cluster of Excellence on Plant Sciences (CEPLAS), Max Planck Institute for Plant Breeding Research, 50829 Cologne, Germany

**Keywords:** Developmental dynamics, Dynamical systems, Modelling, Signalling, Waddington landscape

## Abstract

Developmental biology seeks to unravel the intricate regulatory mechanisms orchestrating the transformation of a single cell into a complex, multicellular organism. Dynamical systems theory provides a powerful quantitative, visual and intuitive framework for understanding this complexity. This Primer examines five core dynamical systems theory concepts and their applications to pattern formation during development: (1) analysis of phase portraits, (2) bistable switches, (3) stochasticity, (4) response to time-dependent signals, and (5) oscillations. We explore how these concepts shed light onto cell fate decision making and provide insights into the dynamic nature of developmental processes driven by signals and gradients, as well as the role of noise in shaping developmental outcomes. Selected examples highlight how integrating dynamical systems with experimental approaches has significantly advanced our understanding of the regulatory logic underlying development across scales, from molecular networks to tissue-level dynamics.

## Teaser

**Summary:** Dynamical systems theory provides a powerful quantitative and intuitive framework to understand developmental processes. This Primer brings key concepts of this framework to the ever-growing community of interdisciplinary developmental biologists.

## Introduction

**Fig. 1.**
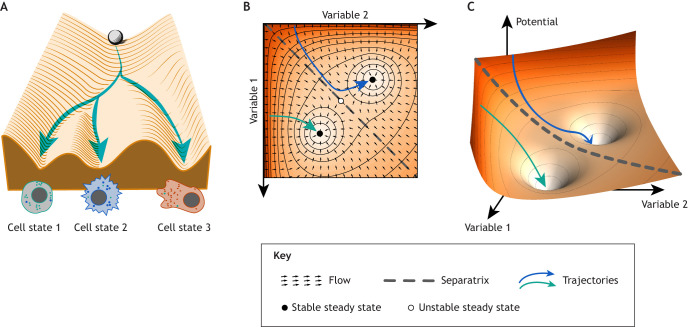
**Visualising phase space representations, steady states and potential landscapes.** (A) Waddington's landscape represents cell fate decisions, with a cell (sphere) traversing downward towards three distinct cell states. (B) Phase portrait depicting interactions of variables, with small arrows showing flow dynamics, the separatrix (dashed line) dividing basins of attraction, and stable/unstable steady states (filled/unfilled circles). (C) Three-dimensional potential landscape representation with two stable states (wells), showing system trajectories (coloured arrows) converging to local minima (white wells). Blue and green arrows in B and C illustrate two system trajectories, starting with different initial conditions.

Decoding the regulatory logic that guides the transformation of a single cell into a complex multicellular organism remains a central challenge in developmental biology. These processes require a tightly controlled sequence of events in which cell division, differentiation and morphogenesis occur in a coordinated fashion in response to positional and temporal cues. Conrad Hal Waddington represented these processes as a ‘landscape’ (see Glossary, Box 1), in which the current state (see Glossary, Box 1) of a cell is represented by a ball initially placed atop a hilly landscape (Waddington, 1957) (Fig. 1A). As if pulled by gravity, this ball rolls downhill representing how development progresses while following the landscape's valleys, which broadly represent the various differentiation pathways available to this system. When the ball arrives at a fork in its path, it will go down one valley and not the other, committing to a developmental fate while leaving other options behind. The topography of this landscape, therefore, determines the developmental potential of the system, including which phenotypes it can achieve and how. The topography of this landscape is determined by the underlying biochemical networks. These networks are remarkably sophisticated, exhibiting properties that defy simple intuition, including multistability (see Glossary, Box 1), which facilitates discrete cell fate decisions, oscillatory dynamics driving rhythmic patterning events, and the ability to integrate spatial and temporal information across scales from molecular gradients and inductive signals.

Box 1. Glossary (mathematical definitions with biological context) **Attractor.** A set of states or a region in state space towards which nearby trajectories of a dynamical system converge or remain within proximity. Usually associated with cell fates.

**Autoactivator.** Generic term used for referring to a gene that promotes its own induction (e.g. when the protein encoded by a given gene can enhance the activity of that gene).

**Basin of attraction.** The range of initial conditions or states that lead to the convergence of trajectories towards a specific attractor in a biological system.

**Bifurcation.** A crucial point or event in the behaviour of a biological system where small changes in conditions or parameters result in a change in the number or stability of attractors in the system. This implies significant qualitative shifts, often associated with changes in pathway activity, developmental trajectories, cellular fate decisions or ecosystem dynamics.

**Bistability/multistability.** Coexistence of two or more than one stable attractor in the phase space. In a deterministic system, the initial condition will determine which attractor the system will follow.

**Canalisation.** The ability of a developmental system to produce a consistent outcome despite environmental or genetic variations.

**Flow.** The set of all trajectories of a continuous dynamical system, describing how points move through phase space over time.

**Genetic oscillator.** Gene regulatory network (GRN) in which genes present sustained oscillations over time.

**Geometric analysis.** A mathematical approach that studies shapes, curves and surfaces of the phase portrait to understand the qualitative behaviour of a dynamical system.

**Hysteresis.** The dependence of a system's current output on its past states, creating a memory effect.

**Continuous versus discrete systems.** Continuous systems evolve smoothly over time with infinitely many possible states, whereas discrete systems change in distinct steps with countable states.

**Initial condition.** Specific state of a system at the starting point of observation, e.g. the concentrations of all relevant biomolecules at time zero.

**Landscape.** A conceptual representation of a system's state across the phase space, often depicted as a topographical map with peaks, valleys and ridges. The height of the landscape can be associated with the potential of an energy landscape or the fitness in an evolutionary system.

**Limit cycle.** An attractive closed orbit in the phase space, also referred to as a periodic orbit. It results in repetitive oscillations over time of the variables involved. It is associated with sustained oscillations in biological processes and requires a negative feedback mechanism.

**Network topology.** The arrangement of interactions (edges) among the components (nodes) forming a network. In a gene regulatory network (GRN), this refers to the set of genes (nodes) and the specific regulatory interactions (edges) between them, taking into account whether those interactions are activating, repressing, or absent. The topology determines how signals and information flow through the network and strongly influences its dynamical behaviour.

**Ordinary differential equations (ODEs).** In dynamical systems theory, a deterministic (i.e. non-stochastic) set of equations that describe the evolution of the variables in the system by specifying their rates of change over time.

**Parameter.** A constant in an equation that represents a specific aspect or property of a biological system (biophysical constants, rates of reactions, environmental factors, etc.) and controls its behaviour.

**Phase portrait.** A geometric representation of all possible trajectories of a dynamical system in its phase space, showing how variables evolve over time.

**Phase space.** Representation of all possible states of a system, with each dimension corresponding to one of the system's variables. Every point in the phase space defines a unique state of the system at a given time, and trajectories in this space depict how the system evolves over time.

**Repellers.** A set of states or a region in state space from which all nearby trajectories of a dynamical system diverge or migrate away.

**Robustness.** The ability of a system to withstand small fluctuations in the variables or parameters of the system without altering the broader trajectory towards an attractor.

**Saddle points.** Crucial points in the phase space of a dynamical system where the behaviour of the system is stable in some directions and unstable in others, resembling a saddle shape in the system's landscape.

**State.** The particular condition (e.g. state of gene expression) or configuration of a biological system (e.g. cell types) at a given moment, typically characterised by the values of its variables.

**Stochasticity.** The presence of randomness in the behaviour or evolution of a dynamical system, arising from internal or external processes.

**Trajectory/orbit.** The path followed by a dynamical system in its state space over time. In a biological system, it describes its temporal evolution.

**Variable.** A dynamic characteristic, property or factor that can be measured or observed.

Dynamical systems theory offers a powerful mathematical framework to capture and analyse this inherent complexity (Strogatz, 2019; Ferrell, 2012). This is achieved by representing the interactions between key variables (see Glossary, Box 1), such as gene expression levels or protein concentrations, as a system of equations, which results in dynamical models that elucidate how changes in these factors over time are encoded by specific rules in the system. Such a quantitative abstraction distils the essence of developmental regulatory networks into tractable models that enable a systematic analysis of their behaviour. These models do not recreate every nuanced aspect of the intricate networks they represent; rather, they illuminate broad regulatory principles. For example, when representing a cell fate decision dependent on a morphogen, the model will incorporate the levels of morphogen and the concentrations of key molecular species involved in the cell fate decision. These species interact through prescribed rules that take into account biophysical or biochemical parameters (see Glossary, Box 1), such as synthesis and degradation rates, diffusion coefficients, or the morphogen concentration threshold to activate their corresponding cell surface receptors. Different modelling approaches may involve distinct levels of granularity, so finer details of the underlying biochemistry responsible for this transduction may not necessarily be included.

For developmental biologists aiming to bridge biological concepts with mathematical models using dynamical systems theory, a series of guiding questions can provide an intuitive entry point (Box 2). These questions explore how variables can switch between states, how outcomes depend on starting conditions, and how oscillatory dynamics and extrinsic signals can be captured. It is important to note that dynamical systems theory is a general mathematical framework that can be applied to a wide range of biological systems beyond developmental biology, such as neuroscience, where it is used to model the dynamics of neural networks; and ecology, where it is employed to study population dynamics and species interactions (Jaeger and Monk, 2014; John et al., 2022; Perez Montero et al., 2024; Rogers et al., 2022; Säterberg and McCann, 2021).

Box 2. How to think about developmental processes as dynamical systems **How can we represent developmental processes quantitatively?**

A dynamical biological system is represented by mutually interacting variables such as concentrations of gene products, their interactions and how those depend on biophysical ‘parameters’, such as transcription/translation and synthesis/degradation rates. By assigning these as measurable variables or constants within one or more equations, it is possible to geometrically plot how changes in each parameter influence the trajectory (see Glossary, Box 1) of the system over time.

**What happens when biological variables influence their own production?**

Consider a gene that promotes its own expression through positive feedback. Depending on the gene's maximum transcription rate and initial protein levels, the system will stabilise at either very low (‘off’) or very high (‘on’) amounts. These stable states, called attractors (see Glossary, Box 1), explain one way in which cells make decisive switches between distinct fates during development. The ability to maintain two stable states emerges when a gene's product strengthens its own expression either directly or indirectly.

**How do broad starting conditions condense to a robust outcome?**

When noise is negligible and no time-dependent signals are involved, initial conditions (see Glossary, Box 1) determine which attractor a system reaches. Each attractor has a ‘basin of attraction’ (see Glossary, Box 1) around it: the set of starting conditions that evolve towards that state. When noise or stochasticity (see Glossary, Box 1) is present, the topography of the landscape (e.g. depth of the attractors) helps explain developmental robustness (see Glossary, Box 1) to random fluctuations. Random fluctuations in gene expression or time-dependent signals can push the system across landscape barriers towards alternative fates.

**How can oscillatory dynamics be captured and understood?**

Oscillatory systems can arise, for instance, from delayed negative feedback on molecular components. As a molecule's concentration increases, it triggers its own inhibition, either directly or indirectly, causing a subsequent reduction in its levels. This weakens the inhibition, allowing its concentration to then rise again, creating cyclical oscillations. The resulting period and amplitude are captured through a dynamical system formalism.

**How can extrinsic signals be incorporated?**

Extrinsic signals can be incorporated as a change of a parameter in the model, and this change can depend on time.

**Fig. 2.**
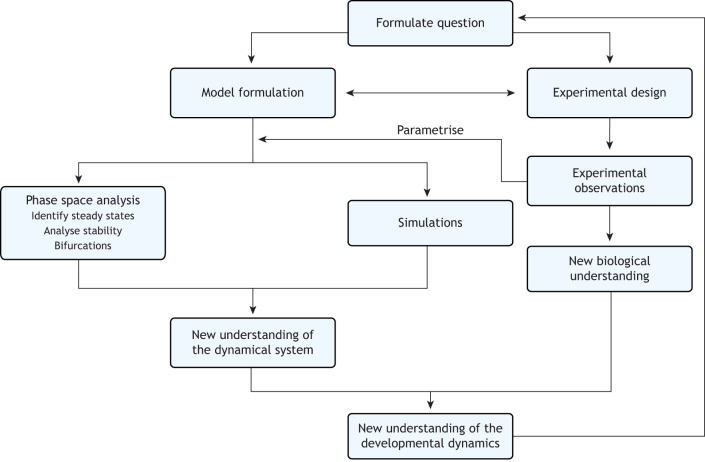
**Key steps for dynamical systems analysis in developmental biology.** Flow diagram showing how experimentalists and modellers collaborate iteratively to develop a dynamical systems model of a developmental process based upon and tested by experiments. A crucial step is formulating the modelling and experimental designs together.

The basis for successfully building a data-driven model of a dynamic developmental process is a good working relationship between modeller and experimentalist that extends all the way from the model formulation and experimental design steps to model validation (Fig. 2). It is commonplace that modellers are approached once the data have been collected and a preliminary model has been formulated. This can sometimes work well but often means that the modeller has to work with what they have been provided instead of working with the best data to model the problem at hand. It is crucial that both parties are involved from the beginning to ensure that the experiments performed are meaningful and provide the information needed for modelling. This is also a training period for both modeller and experimentalist, as both must understand the limitations and feasibility of the experiments and the modelling, respectively. For instance, validation of a data-driven model of a dynamic developmental process places high demands on the experimentalist. Dynamical modelling requires time series data with high temporal resolution, as this allows the system's dynamics to be captured more accurately and enables a more precise model to be formulated. Also, perturbations to challenge the model should be performed with high resolution and with ranges of drug concentrations. While the experimentalist has to understand the importance of data collection for increasing the model accuracy, the modeller has to comprehend the constraints and intricacies of the experimental system and the amount of work that goes into collecting the data that they are requesting. Thus, the process of building a dynamical systems model is iterative in nature, requiring constant feedback between modeller and experimentalists and multiple rounds of modelling and experiments.

This Primer aims to elucidate how five core dynamical systems concepts – phase space, bistable switches, stochasticity and time-varying systems, which we discuss together, and oscillations – can provide a unified conceptual framework for decoding developmental patterning logic across scales, while highlighting the power of dynamical systems theory in capturing and analysing complex biological phenomena. We also provide interactive Python code to the reader who wishes to investigate the presented concepts further (see supplementary information).

## (1) Phase space: the dynamics of cell fate trajectories and robustness

At the heart of dynamical systems theory lies the concept of ‘phase space’ (see Glossary, Box 1), a multidimensional abstract space where each axis represents a system variable, such as transcription factor concentrations, mRNA levels or the abundance of other relevant biomolecules. For instance, in a simple two-gene network, the phase space could be a two-dimensional (2D) plane with axes representing the concentrations of the two interacting components (variables 1 and 2 in Fig. 1B). The ‘state’ of the system at any given time is represented by a point in this phase space. The ‘initial condition’ refers to the specific state of the system at the starting point of observation, determining where in phase space the system begins its evolution. Given an initial condition and the underlying flow, a unique trajectory of the system can be determined. As the system evolves over time, the point traces out a trajectory reflecting the changing concentrations of the biomolecules (Strogatz, 2019; Manser and Perez-Carrasco, 2024). This is represented in Fig. 1B in which blue and green arrows indicate two different resulting trajectories starting at different initial conditions.

To visualise and analyse the system's behaviour, we employ phase portraits (see Glossary, Box 1), which are graphical representations of the flow (see Glossary, Box 1) in the phase space. The flow consists of vectors indicating the rate of change of the system at every point (Fig. 1B, small arrows), determined by the underlying structure of the gene regulatory network (GRN), typically formulated as a system of ordinary differential equations (ODEs; see Glossary, Box 1). These ODEs capture the biochemical kinetics of the network, such as the rates of transcription, translation, degradation and regulatory interactions between genes. By plotting the flow vectors in the phase space, we can identify key features of the system's dynamics, such as steady states and the trajectories leading to them.

Steady states, or fixed points, are equilibrium points where the flow is zero, and the system remains unchanged. These steady states can be classified through stability analysis as stable attractors (Fig. 1B, filled circles), which draw nearby trajectories inward over time; unstable repellers (see Glossary, Box 1) and saddle points (see Glossary, Box 1) (Fig. 1B, unfilled circle), which push nearby trajectories away in at least one direction. The stability of a steady state is determined by its response to small perturbations. For instance, if a system returns to its original steady state after a small perturbation in any direction, the steady state is considered stable. Stable attractors are of particular interest in developmental biology because they represent the discrete cellular phenotypes arising from the underlying GRN. This is essential for the concept of ‘canalisation’ (see Glossary, Box 1), which refers to the ability of a developmental system to produce a consistent outcome despite environmental or genetic variations (Hallgrimsson et al., 2019; Panfilio and Roth, 2013; Siegal and Bergman, 2002). In the context of GRNs, canalisation arises from the network topology (see Glossary, Box 1), which constrains the system's dynamics and ensures that the steady states remain stable and attractive over a wide range of conditions.

The ‘basin of attraction’ surrounding a stable attractor encompasses all initial states that will eventually converge to that attractor, regardless of the starting conditions within the basin. This concept is visually represented in Fig. 1A, which shows Waddington's epigenetic landscape, envisioning the developmental process as a cell (ball) rolling down a landscape with hills and valleys representing distinct differentiation pathways. In this metaphor, the three basins of attraction correspond to the valleys, and the stable attractors represent the lowest points of these valleys. In Fig. 1B, two basins are separated by a separatrix (the boundary between the two basins, dashed line), and the blue and green arrows correspond to trajectories with initial conditions located in each one of the basins of attractions. Similarly, Fig. 1C shows them as valleys in the potential landscape. The landscape's topography dictates the system's developmental potential, determining the accessible phenotypes; the slope of the landscape dictates the speed of change, and the depth of the valleys reflects the resilience of a developmental pathway against perturbations and noise.

Noise, or stochasticity, i.e. fluctuations in gene expression, is another important factor that can influence the behaviour of cellular dynamics (Box 3). In biological systems, noise can arise from various sources, such as the inherent stochasticity of biochemical reactions, variations in the cellular environment, or external perturbations (Ladbury and Arold, 2012; Tsimring, 2014). While noise can introduce variability in gene expression patterns, the architecture of GRNs sometimes seems to have evolved to be robust to these fluctuations (Filippi et al., 2016; Uda et al., 2013). The concept of robustness refers to the ability of a system to maintain its function or output despite perturbations or noise. In the context of GRNs, robustness is often achieved through feedback loops, redundancy and the presence of stable, ‘deep’ steady states. Noise can be implemented using different formalisms, ranging from an exact and discrete approach – by enumerating the biochemical reactions, which can be simulated numerically using the Gillespie algorithm – to an approximate and continuous approach such as the chemical Langevin equation, which is suitable when noise levels are less pronounced (Gillespie, 2000, 2007; Wilkinson, 2009).

Box 3. Impact of noise on dynamical systems in development **Attractor stability and transitions**

Noise can perturb the state of the system in the ‘phase space’, changing the ‘landscape’ and potentially pushing the cell out of the current ‘basin of attraction’, triggering transitions between developmental states (Coomer et al., 2022). This occurs in bistable and multistable systems, where noise can induce switching and influence the transitions between states. This has implications for understanding cell fate decisions in complex developmental landscapes, such as during stem cell differentiation.

**Bifurcations**

Near bifurcation points (see Glossary, Box 1), small fluctuations can lead to qualitative shifts in system behaviour, potentially inducing abrupt fate changes during development. For instance, close to bifurcations, noise can highly impact the signals at which cell transitions are observed and their timing (Dalwadi and Pearce, 2023; Perez-Carrasco et al., 2016; Weber and Buceta, 2013).

**Emergence of noise-induced patterns**

Stochastic fluctuations can give rise to spatial or temporal patterns that are absent in deterministic models (Buceta et al., 2003). This may contribute to the observed variability and robustness in developmental patterning processes (Aoki et al., 2013; Kellogg and Tay, 2015).

**Signal amplification via stochastic resonance**

In some developmental contexts, noise can counterintuitively enhance the system's sensitivity to weak signals, a phenomenon known as stochastic resonance, which might play a role in growth (Bhalerao, 2024; Meroz and Bastien, 2014) and in precise spatial patterning (Perez-Carrasco et al., 2018).

**Temporal coordination**

In oscillatory systems, such as the segmentation clock, noise can affect the synchronisation of genetic oscillators (see Glossary, Box 1) across cell populations, influencing the precision of periodic patterning events (Delaune et al., 2012; Horikawa et al., 2006; Riedel-Kruse et al., 2007).

Understanding these noise-induced phenomena is crucial for developing more realistic dynamical models of developmental processes and for explaining the robustness and variability observed in biological systems.

Analysis of phase portraits provides a powerful tool for understanding the canalisation and robustness of GRNs (Lapidus et al., 2008; Manu et al., 2009; Panfilio and Roth, 2013). By examining the topology of the phase space, we can identify the basins of attraction, the stability of steady states, and the transitions between them (Jaeger and Monk, 2014). Thus, geometric analysis (see Glossary, Box 1) reveals the dynamical repertoire of a GRN, which refers to the collection of qualitatively different behaviours that the system can exhibit under different conditions. In addition, by varying the parameters of the equations representing the GRN, we can explore how the topology of the phase space changes, identifying ‘bifurcations’ – points at which small parameter changes can transform one stable pattern into another or cause steady states to emerge or disappear. This systematic exploration of parameter space can be visualised through bifurcation diagrams, which map out how the number and stability of steady states change as parameters are varied, providing a comprehensive view of a system's dynamical repertoire across different conditions (Salvi et al., 2016). A detailed example of such bifurcation analyses is provided in the context of bistable switches and oscillators in the following sections. This analysis can provide insights into the plasticity of the developmental system, revealing how different environmental signals or genetic perturbations can alter the landscape of cell fates and drive the system towards alternative developmental trajectories.

Besides the qualitative investigation of phase portraits, more quantitative geometric analysis can also provide information on the robustness established by GRNs. The size and shape of the basins of attraction (Fig. 1C) provide information about the robustness of the corresponding cell states. Large, deep basins indicate highly stable and robust states, whereas shallow or narrow basins suggest states that are more sensitive to perturbations and noise. Moreover, the presence of boundaries between basins can ensure that transitions between states occur only in response to specific signals or perturbations, thus maintaining the integrity of the developmental process (Coomer et al., 2022; Exelby et al., 2021).

Analysis of the phase space can also be complemented with numerical simulations to obtain trajectories of the dynamical system that can be compared with experiments.

### Biological examples

In embryonic stem cells in vertebrates, high-dimensional GRN models have revealed attractor landscapes with basins corresponding to the pluripotent and differentiated states. These models capture how the core pluripotency factors, such as OCT4 (POU5F1), SOX2 and NANOG, interact to maintain the pluripotent state, while perturbations to this network can trigger differentiation trajectories (Hovland et al., 2022; Pfeuty et al., 2018). Models incorporating additional regulatory factors, such as FGF4 and GATA6, and external application of signalling molecules, such as LIF, have further elucidated the complex dynamical landscape underlying embryonic stem cell fate decisions, revealing how multiple feedback loops and regulatory circuits shape the stability and accessibility of different cell states (Brackston et al., 2018). Similarly, in the CDC25-WEE1 system, which governs cell cycle progression, dynamical models expose the attractor basins of quiescence and proliferation, demonstrating how extracellular signals can modulate the stability of these states by altering the balance of CDC25 and WEE1 activities (Li and Wang, 2014). Geometric analysis of phase portraits enables the visualisation and quantification of these developmental dynamics, providing a rigorous yet intuitive approach to decode the regulatory logic that transforms genetic networks into robust cellular identities.

## (2) The bistable switch: binary cell signalling decisions

The bistable switch is a dynamical system, such as a single cell or pathway, that can transition between two stable, steady states. Bistable switches govern numerous binary cell fate decisions during development, including stem cell differentiation and cellular reprogramming (Chickarmane et al., 2006; Graf and Enver, 2009; Graham et al., 2010). For instance, the mutual inhibition between transcription factors driving alternative lineages can create bistability (see Glossary, Box 1), with each stable attractor representing a distinct cell identity or fate (Alon, 2007). The system's initial conditions, such as the levels of specific transcription factors, determine which of the two stable states the cell will converge to and commit to a particular developmental pathway.

**Fig. 3.**
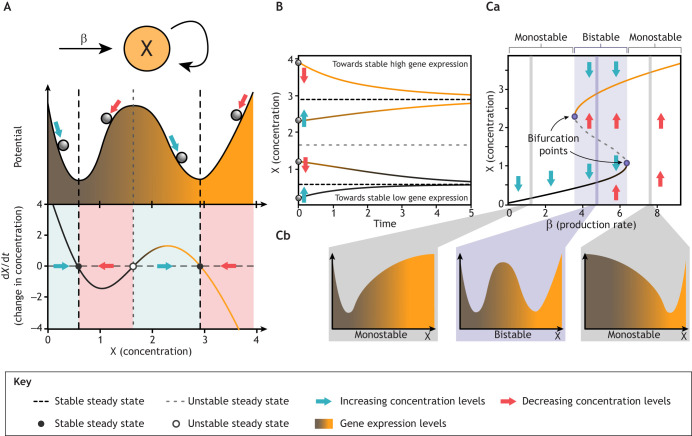
**Dynamical systems analysis of bistable gene regulation.** (A) Top: Simple autoregulatory circuit where protein X is constitutively expressed at rate β and autoactivates itself, increasing the production rate (see Eqns 1 and 2). For the sake of simplicity, degradation is not represented. Bottom: Schematic of the potential landscape and phase space representation showing two stable steady states (minima of the potential/black circles in the phase space) separated by an unstable steady state (maximum of the potential/white circle in the phase space). Coloured arrows indicate directions of state changes, pointing towards stable steady states. Orange gradient represents gene activation levels. (B) Time evolution of the system from different initial conditions (grey spheres), showing that trajectories evolve away from the unstable state (grey dashed horizontal line), converging to either high or low stable steady states (black dashed horizontal lines). (Ca) Bifurcation diagram revealing system behaviour as the constitutive production rate β varies. The shaded region indicates a bistable regime bounded by bifurcation points (purple circles) where the system transitions between monostable and bistable regimes. In this panel, solid lines (yellow, black) represent stable steady states; the grey dashed line shows the unstable steady state. Blue/red arrows indicate trajectory directions. (Cb) Schematic indicating qualitative changes in potential landscape as β increases from left to right, showing the transition from monostable (single well) through bistable (double well) back to monostable behaviour. Analysis and simulations correspond to the systems described in Eqn 2. Code for reproducing this figure, along with the parameter values of the model, can be found in the supplementary information.

A simple example that illustrates a bistable switch is a transcriptional autoactivator (see Glossary, Box 1), which we discuss in detail here to exemplify the application of dynamical systems theory in biological research. In a transcriptional autoactivator, a transcriptional activator, *X*, is produced (transcribed and translated) under the control of *X* itself, either directly or indirectly (Fig. 3A, top). The intuitive concept for a bistable switch is that of a dynamical landscape with two basins of attraction separated by a barrier, as shown in the potential landscape (Fig. 3A, middle). Under this description, the system flows into either of the two steady states, depending on the initial conditions. The transition between the two steady states, termed ‘the switch’, can occur due to external noise or changes in the underlying regulatory network. Significant external noise, such as fluctuations in gene expression or signalling, can perturb the system enough to push it over the boundary separating the two states, causing it to transition to the alternative steady state. Conversely, changes in the parameters governing the regulatory network, such as alterations in transcription factor levels or binding affinities, can modify the epigenetic landscape, potentially eliminating one or more of the stable steady states and forcing the system to transition to the remaining attractor states.

For simplicity, we consider a simple autoactivation circuit that can be described by a one-dimensional dynamical system (Scott et al., 2007; Tyson et al., 2008). The variable in the system is the concentration of the transcriptional activator *X* and its dynamics can be described by the balance of production and degradation, following the equation:
(1)

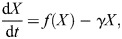
where *f*(*X*) describes the rate of production of *X* (for simplicity, transcription and translation are combined), which depends on *X* itself; and *γX* is a linear degradation term with a degradation rate *γ*. Since *X* is an activator, *f*(*X*) should be an increasing function of *X*. For the current example, we assume that *f*(*X*) has the form:
(2)

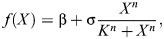
where β is a constitutive production rate and *σ* is the maximal increase in production rate due to the autoactivation. The autoactivation is represented by a Hill function term, where *K* is the concentration at which the Hill function adopts half its maximal value, and *n* is the Hill coefficient, controlling the slope of the Hill function. As a result, the production rate *f*(*X*) follows a sigmoidal function with minimal production *β* in the absence of *X*, and saturates at β+σ in abundance of *X*.

The dynamics of variable *X* will be determined by the values of the constant parameters β, γ, σ, *K* and *n*. For instance, the steady states of the system can be found by calculating the values of *X* at which the flow vanishes 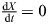. This is satisfied when *f*(*X*)=*γX* (see Eqn 1). For certain parameters, this equation can result in three steady states, which can be easily visualised graphically (Fig. 3A, black and grey dashed lines). These steady states can be categorised as stable and unstable steady states (corresponding to the minima and maximum, respectively, of the potential shown in Fig. 3A).

The stability of the steady states can be formally calculated using perturbation theory, but can be conveniently analysed graphically by plotting the rate of change 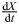 as a function of *X*. The graph for the case of the autoactivation circuit is shown in Fig. 3A (bottom), where *f*(*X*)−*γX* is plotted as a function of *X*. The crossing points of this function with the *X* axis are the steady states (shown as circles). Whenever the function is positive, it means that *X* is increasing with time (since 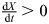). Whenever the function is negative, it means that *X* is decreasing with time (since 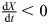). One can, therefore, track the flows in the system in different regimes (marked by arrows). We can conclude from these flows that the lowest and highest steady states are stable (the flows are pointing towards the steady states from both sides), whereas the middle steady state is unstable (the flows are pointing away from the steady state). Thus, the graphical analysis shows that there are two stable steady states corresponding to low and high *X* values, and another steady state between them, which is unstable.

The time evolution trajectories shown in Fig. 3B demonstrate how, depending on the initial condition, the concentration of *X* will end up in the high or low steady state (black dashed lines). In particular, initial concentrations of *X* above the unstable state (grey dashed line) will evolve to the stable steady state with high *X*, while those below the unstable state will evolve to the low *X* steady state. Hence, the system has two basins of attraction separated by the unstable state.

Finally, we can ask how the steady states and their stability change as we vary the parameters (i.e. the landscape) of the system. This can be visualised in a bifurcation diagram (Fig. 3Ca), which in this case shows how the system exhibits different regimes as we vary the fixed maximal production rate parameter β. The diagram maps the behaviour of the system across a relevant parameter range and shows where the bifurcations occur (purple circles). It also shows another emergent property of bistable systems: ‘hysteresis’ (see Glossary, Box 1) or irreversible behaviour with varying parameter β. If one starts at low β values and increases it, the system will remain at the low steady state until it reaches the right bifurcation point and then switch to the high steady state. However, if one starts at high β values and decreases it, the system will remain at the high steady state until it reaches the left bifurcation point and only then switch back to the low steady state.

These transitions can be visualised through the potential landscapes in Fig. 3Cb. If we start with a low β value, we only have a single steady state at a low *X* value (left). As we increase β, at a specific parameter value the system will bifurcate and exhibit three steady states (two stable and one unstable; as in Fig. 3A, middle). Increasing β further results in another bifurcation, where the system switches back to a single steady state, this time at high *X* values (right). Thus, simply by changing one parameter, the dynamical landscape of the system changes, dramatically affecting the dynamics of the system. For the case of a bistable switch, changing the constitutive production can take the system from one low *X* state, to two co-existing low and high *X* states, to one high *X* state. The system exhibits hysteretic or irreversible behaviour depending on the previous state of the system and the direction in which the parameter β is changing. It is worth noting that the type of bifurcation described here is known as a saddle-node bifurcation (Strogatz, 2019). Other types of bifurcations will be introduced in the following sections.

A similar analysis can be performed on positive feedback circuits that contain more than a single gene. For example, a classical two-gene circuit that can form a bistable switch is the mutual inhibition circuit where genes *X* and *Y* mutually inhibit each other. This mutual inhibition forms a positive feedback circuit because the inhibition of an inhibition results in an activation. A 2D dynamical systems analysis extends the same concepts discussed here to flows in 2D landscapes (Cherry and Adler, 2000; Gardner et al., 2000). More generally, any feedback loop involving only positive interactions, or an even number of negative interactions, introduces positive feedback, and can give rise to bistability.

### Biological examples

In vertebrates, the interplay between core pluripotency factors such as OCT4, SOX2 and NANOG forms a regulatory network that contains a positive feedback loop, driving to a bistable switch in embryonic stem cells, with the pluripotent state representing one stable attractor and differentiation towards specific germ layers (endoderm, mesoderm and ectoderm) corresponding to alternative attractors (Chickarmane et al., 2006; Giri and Kar, 2024). Morphogen gradients can induce bifurcations that destabilise the pluripotent attractor, driving cells towards these lineage-specific attractors (Brown et al., 2011). Similarly, in *Arabidopsis*, the vernalization process, in which the cold exposure is epigenetically stored to regulate flowering time, involves a bistable switch in the expression of the floral repressor FLOWERING LOCUS C (FLC) at the single-cell level (Angel et al., 2011; Satake and Iwasa, 2012), enabling an irreversible transition to flowering upon sensing of the appropriate seasonal cues. By encoding developmental potential as discrete attractor states and facilitating precise, irreversible fate transitions through parameter changes or signals, bistable switches provide a fundamental mechanism for cell fate determination and lineage commitment during embryogenesis.

## How can a system move between stable states? (3) Stochasticity and (4) time-dependent dynamical systems

**Fig. 4.**
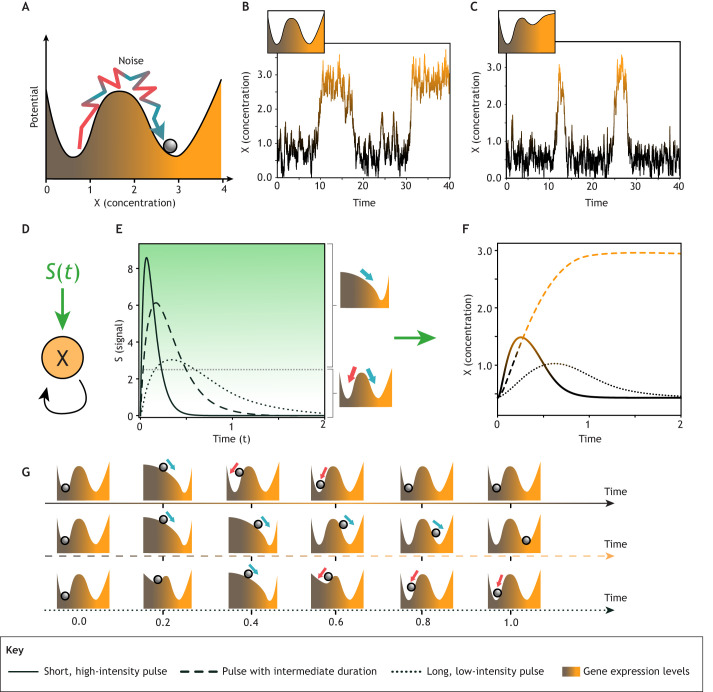
**Dynamics of stochastic and time-dependent bistable switches.** (A) Potential landscape showing noise-induced transitions between stable states, where stochastic fluctuations can drive the system over the barrier. (B,C) Stochastic simulations showing switching between low (black) and high (orange) expression states under different noise intensities for two different potentials. Insets at the top are cartoons of the potential; in C, the higher stable state is shallower, making the system spend less time in it. (D-G) Analysis of signal-induced state transitions. (D) Circuit diagram showing autoactivating gene *X* regulated by time-varying signal *S*(*t*). For the sake of simplicity, degradation is not represented in the diagram. (E) Different pulses of input signal *S*(*t*) that modify the potential landscape (insets on the right). Horizontal dashed line represents the signal level at which the system switches from being bistable (below the line) to monostable (above the line). (F) Numerical simulations of the resulting gene expression trajectories show how different signal profiles lead to distinct final states for the autoactivating switch (Eqn 4). (G) Schematic of the time evolution of the system state (grey sphere) in response to the three different signal profiles presented in E and F (line styles of the time axes represent the same as in E,F), demonstrating how temporal signal dynamics influence transition paths between states. Code for reproducing this figure, along with the parameter values of the model, can be found in the supplementary information.

Stochasticity and time-dependent signals can both allow the system to move between steady states. The effect of stochastic fluctuations on bistable systems can be visualised through a potential landscape representation (Fig. 4A), where noise can drive transitions from one stable state to another. These noise-induced transitions manifest as switching between low and high expression states over time, as demonstrated by different stochastic trajectories (Fig. 4B,C).

External signals can also cause systems to move between steady states. Developmental processes are often modulated by biochemical signals and environmental cues, referred to as signals hereafter. Such modulation can be phenomenologically captured by effective parameters of the underlying regulatory network that change over time, which therefore confers a time-dependent nature to the underlying dynamical system. When the timescales associated with the signals are comparable to the characteristic timescale of the developmental process, this time-dependent nature of the dynamical system becomes more relevant, and transient, non-trivial dynamical behaviours can arise (Morelli et al., 2009).

Signals can be simulated as time-varying parameters that either change over time in a discrete (two or more distinct levels) (Perez-Carrasco et al., 2018; Verd et al., 2014) or continuous (see Glossary, Box 1) fashion (Nandan and Koseska, 2023; Palau-Ortin et al., 2015; Verd et al., 2019). Discrete signals change in discontinuous steps, such as transcriptional regulators being active or inactive. A continuous signal would be, for example, a morphogen gradient changing concentration levels gradually over space and time. When the dynamical system's equations explicitly depend on time itself, not just the state variables such as gene expression levels, the system is classified as non-autonomous. Another option to incorporate signals in a model is to have the signal as a modelled variable (Abley et al., 2021), so the system does not explicitly depend on time. In both cases, one can obtain an ‘instantaneous phase portrait’ of the dynamical system throughout the studied time window by computing the phase portrait at the values the signal acquires at specific times of the simulation.

As an example, Eqn 1 can be turned into a time-dependent bistable switch, such as:
(3)

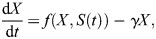
where *f*(*X*, *S*(*t*)) is now defined as:
(4)

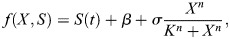
and *S*(*t*) is the signal that changes over time (Fig. 4D). Compared with the autonomous autoactivator (Eqn 2), we can see how, as *S*(*t*) changes in time, the phase portrait changes accordingly. Therefore, the state of the system, *X*(*t*), will experience a phase portrait that changes over time, affecting its trajectory. Previous theoretical work has proposed a classification of transient behaviours that can arise in a time-dependent toggle switch model (Verd et al., 2014). The most common and well-studied behaviour is the shift of the system from one attractor to another when *S*(*t*) induces a bifurcation, destabilising the steady state of the system.

Different temporal profiles of the input signal *S*(*t*) will alter the potential landscape in distinct ways, leading to varying and sometimes unexpected gene expression trajectories (Koch et al., 2024; Verd et al., 2014). For instance, in Fig. 4E-G, we show how a transient signal pulse can regulate the switching of *X* from a low to a high state. In this case, a short, high-intensity pulse or a long, low-intensity pulse fail to induce switching, whereas a pulse with intermediate duration and intensity successfully activates gene *X.* This dependency of the system trajectory on the input signal is another example of hysteresis, whereby the system behaviour depends on its own history.

### Biological examples

The temporal modulation of bistability, coupled with stochastic fluctuations in the network components, provides a powerful mechanism for orchestrating precise yet flexible developmental transitions, elucidating the mechanisms underlying the robustness and adaptability of biological systems. In vertebrate neural tube patterning, the dorsoventral gradient of sonic hedgehog (Shh) activates the Gli pathway, which exhibits pulse-like signalling that controls the spatiotemporal patterning of neural progenitors (Balaskas et al., 2012; Cohen et al., 2014). In the same system, the GRN downstream of Gli maximises barriers between steady states to prevent cell-type switching inside each neural progenitor domain, maximising the precision in the patterning of the tissue (Exelby et al., 2021). In *Arabidopsis* seed germination, the regulatory network involves the abscisic acid (ABA) and gibberellic acid (GA) hormones, forming coupled positive feedback loops that create a bistable switch between non-germination and germination states (Abley et al., 2021; Topham et al., 2017). An increase in GA biosynthesis during seed sowing alters the phase portrait, destabilising the non-germination state. It has been proposed that this developmental signal, together with the intrinsic stochasticity of the GRN, is a plausible model to understand variability of germination time across different *Arabidopsis* accessions (Abley et al., 2021).

## (5) Oscillatory dynamics: orchestrating periodic patterning

**Fig. 5.**
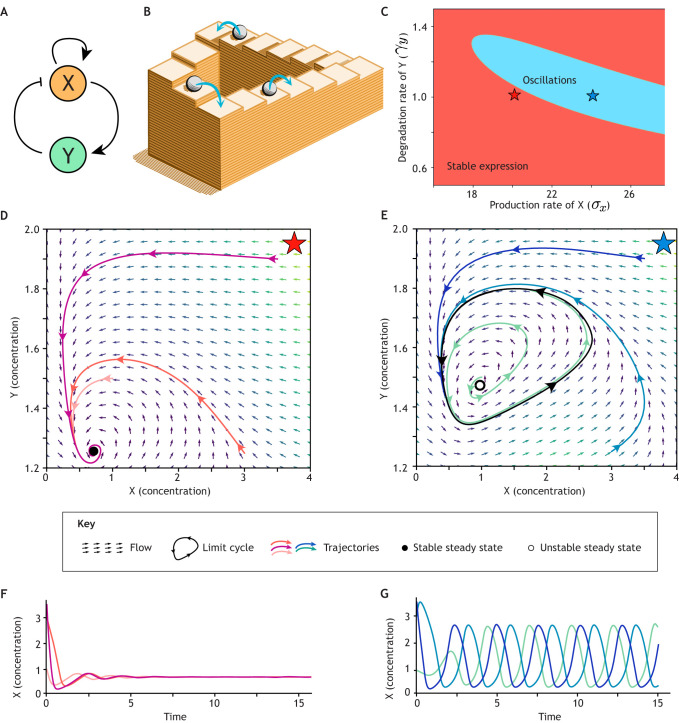
**Dynamical system exhibiting oscillatory behaviour.** (A) A gene regulatory network capable of oscillations, corresponding to Eqns 5 and 6. (B) Sustained oscillations cannot be directly conceptualised as a particle rolling down a landscape. Instead, they can only be represented through optical illusions, such as the Penrose stairs. (C) Bifurcation diagram in a 2D parameter space showing that the resulting dynamical behaviour depends on the parameters of the system, classified as sustained oscillations (blue) or stable steady constant expression (red). For the sake of simplicity, constitutive production and degradation are not represented. Stars represent parameter sets used in D-G. (D) Phase portrait outside the oscillatory regime; a single attractive steady state (black circle) attracts the trajectories independently of the initial condition (each coloured line belongs to a different initial condition). (E) Phase portrait in the oscillatory regime, the steady state is now repulsive (unfilled circle), and a limit cycle (black line) attracts all the trajectories independently of the initial condition (each coloured line belongs to a different initial condition). (F,G) Resulting trajectories in time of the expression of gene *X* for the same conditions shown in D and E, respectively. Code for reproducing this figure, along with the parameter values of the model, can be found in the supplementary information.

In addition to evolving towards a stable steady state, there are other possible behaviours of dynamical systems; this is the case for genetic oscillators. In a genetic oscillator, gene expression patterns change in a periodic manner over time without ever reaching a fixed steady gene expression (Monk, 2003). An example of a GRN that oscillates is shown in Fig. 5A, consisting of an activating transcription factor *X* and a repressing transcription factor *Y*. Following the same logic as before, we can write a set of ODEs that describe the regulatory interactions as increasing/decreasing functions of *X* and *Y*, respectively (Guantes and Poyatos, 2006):
(5)

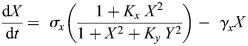

(6)

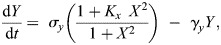
where *σ*_*x*_ and *σ*_*y*_ are the basal production rates of *X* and *Y*, respectively; *K_x_* and *K_y_* control the interaction strength, and γ_x_ and γ_y_ are degradation rates.

If we look at the oscillatory trajectories in the phase space, they form closed orbits that receive the name of ‘limit cycles’ (see Glossary, Box 1) (Strogatz, 2019). Stable limit cycles possess analogous attracting properties to steady states, attracting trajectories starting from different initial conditions. It is interesting to realise that our Waddingtonian landscape intuition starts to fail in this scenario, since it is impossible to have a ball rolling downhill continuously and return to the initial state, as illustrated by the Penrose stairs in Fig. 5B. This reaffirms the power of the ODE description, which does not assume any landscape other than stating the regulatory logic of the circuit.

Similar to a bistable switch, the system's behaviour is sensitive to changes in its parameters, which can affect the emergence of oscillations, as shown in the bifurcation diagram in Fig. 5C. This diagram highlights how non-intuitive combinations of parameters control the behaviour of the system, which can exhibit either a single, stable, attractive steady state (Fig. 5D), or an attractive limit cycle surrounding a repeller (Fig. 5E). The resulting time trajectories demonstrate how the system either converges to a steady state (Fig. 5F) or develops sustained oscillations (Fig. 5G).

The emergence of oscillations can be understood intuitively. In the proposed network (Fig. 5A), when the expression of gene *X* rises, it sequentially leads to an increase in the expression of gene *Y*. As the expression of gene *Y* builds up, it will feed back into gene *X*, repressing its expression. Subsequently, with the suppression of gene *X*, gene *Y* is no longer activated, which then allows the expression of gene *X* to increase again. This cycle reinstates the oscillatory pattern. Importantly, oscillations depend on an appropriate balance between the timings of activating and repressive interactions in the network. In the example, the autoactivation of gene *X* plays a crucial role in this balance; in other models, delays in the negative feedback between *X* and *Y* are required to generate oscillations, which are therefore promoted in feedback loops with several intermediate steps (Lev Bar-Or et al., 2000; Page and Perez-Carrasco, 2018; Takashima et al., 2011). For example, a well-known genetic oscillator that employs a delayed repression, the repressilator, operates through a ring network of three genes that sequentially repress each other (Elowitz and Leibler, 2000). The introduction of more intermediate species increases the complexity of the system, leading to a larger number of parameters. In such cases, this delay can be effectively incorporated into the description of the regulatory network using delay differential equations, which include the delay explicitly in the flow of the dynamical system (Monk, 2003). Models of genetic oscillations show clearly the importance of understanding the timescales of interactions as well as their activating or repressive natures.

Finding limit cycles, analytically or numerically, is more challenging than finding steady states. This complexity arises because limit cycles do not follow the steady state condition d*X*/d*t*=d*Y*/d*t*=0. However, they can be identified through numerical integration of the ODE system. Moreover, if we were to search for steady states in Eqns 5 and 6, we would find that inside the oscillating limit cycle trajectory, there exists an unstable steady state acting as a repeller (Fig. 5E). This repeller pushes trajectories away from it and towards the surrounding stable limit cycle (Fig. 5E,G). The presence of this repelling steady state paired with the stable oscillating limit cycle, together known as a repeller-cycle pair, is a common feature found in dynamical GRN models exhibiting oscillatory behaviours.

Similar to the bistable switch, we can tune external parameters to change the dynamical regime of the system. For instance, in our case, we can drive a system out of the oscillatory state by changing the production rate of gene *X* (Fig. 5C). During this transition, we would observe how the limit cycle shrinks and collides with the repeller, leading to an ordinary stable steady state. This is another example of bifurcation, called Hopf bifurcation (Strogatz, 2019), and is one way by which oscillations emerge or vanish. In addition, limit cycles can exist in more intricate landscapes, as occurs in the AC/DC genetic circuit (Perez-Carrasco et al., 2018), a GRN found in the gap gene network in *Drosophila* and in vertebrate neural tube dorsoventral patterning. This landscape mirrors a bistable switch but with a crucial difference: one of the steady states is substituted by a cycle.

### Biological examples

In *Drosophila* neurogenesis, a genetic oscillator driven by the transcription factors Hunchback (Hb) and Krüppel (Kr) couples with Notch signalling components to create a ‘salt-and-pepper’ pattern of neuroblasts (Li et al., 2013). Similarly, the segmentation clock, essential for periodic somite formation during vertebrate embryogenesis, is driven by the oscillatory expression of cyclic genes regulated by Notch, fibroblast growth factor and Wnt signalling pathways (Dequéant et al., 2006; Niwa et al., 2011; Sonnen et al., 2018). The interplay between these oscillators and morphogen gradients determines the timing and spatial organisation of developmental events (Dubrulle and Pourquié, 2004; Yamanaka et al., 2023). By modelling these oscillatory systems using coupled differential equations, researchers can explore the feedback loops, time delays and spatial coupling mechanisms that give rise to robust and synchronised oscillatory behaviour, providing insights into the complex dynamics underlying pattern formation and developmental transitions (Cooke and Zeeman, 1976; François and Mochulska, 2024; Klepstad and Marcon, 2024; Morelli et al., 2009).

## Further material

We provide interactive Python code to allow the reader to investigate further the presented dynamical systems (see supplementary information). Specifically, the code is presented in the form of two Jupyter notebooks, one for the bistable switch, and one for the oscillations. For readers less familiar with Python and coding, we have also implemented these Jupyter notebooks as applets using the Google Colab platform, which does not require prior coding experience:

- Bistable switch Colab notebook: https://colab.research.google.com/drive/1u8kRrqaWmc5utEgJKiu0yohfaD8nd4PU?usp=sharing;
- Oscillations Colab notebook: https://colab.research.google.com/drive/1IMMwyNg0d2FkAzNL_tLBOYgkPbYjQFvz?usp=sharing.

To run the applets with Colab, load each applet in the web browser and press the triangles on the left to run each segment of the code in the browser. Be sure to run the code in order from top to bottom. The code will generate interactive graphs in which parameters can be adjusted.

## Conclusion

The examples highlighted throughout this Primer illustrate the immense power of dynamical systems theory for decoding the regulatory complexity underlying embryogenesis and multicellular patterning. By abstracting the intricate molecular networks into quantitative models amenable to mathematical analysis, this approach has yielded key insights into how gene regulatory dynamics facilitate pivotal developmental processes, such as cell fate specification, rhythmic patterning, and irreversible fate transitions.

Notably, the ability to visualise and analytically characterise the epigenetic landscapes constraining cellular trajectories has illuminated foundational principles. The existence of discrete attractor states corresponding to specific cell fates or oscillatory patterns accounts for the discreteness and robustness observed experimentally. Crucially, dynamical models elucidate how the architecture of regulatory networks encodes this patterning potential through features such as multistability, oscillations and signal integration, which allows the bridging of genotypes to phenotypes in a predictive framework.

As experimental techniques rapidly advance, the quantitative data obtainable from developmental systems is becoming increasingly high-resolution and comprehensive. Live-imaging approaches can now be used to visualise dynamic reporters in space and time, enabling accurate characterisation of gene expression dynamics, morphogen gradients and cellular behaviours during embryogenesis. Coupled with advanced image analysis pipelines, this capability holds potential for unprecedented detailed mapping of developmental trajectories and attractor landscapes governing cells as they navigate bifurcation points. In parallel, genomic and single-cell technologies are unveiling regulatory architectures at ever-finer resolution, facilitating the construction of predictive dynamical models for diverse patterning phenomena.

The quantitative power of dynamical landscapes has also enabled new modelling approaches in which, rather than deriving the landscape from a detailed set of biochemical reactions, the landscape is inferred directly from observed dynamics. In these approaches, the inferred landscape becomes the foundational element of the model and allows the use of the same suite of dynamical systems theory tools discussed in this Primer (Camacho-Aguilar et al., 2021; Corson and Siggia, 2012, 2017; Corson et al., 2017; Raju and Siggia, 2024; Sáez et al., 2022). For many biological systems, this approach can be used to represent the form of the dynamics formally on a 2D landscape that captures the behaviour of the associated higher-dimensional dynamical system model (Rand et al., 2021).

However, despite these opportunities, significant challenges remain in leveraging dynamical systems theory to unravel developmental programmes. Model construction remains laborious, requiring comprehensive knowledge of underlying network components and their regulation. Scalability to higher dimensions poses analytical and computational hurdles. Systematic approaches integrating experimental data-driven modelling with mathematical analysis frameworks are needed. Additionally, many developmental processes remain inadequately captured in deterministic ODEs, necessitating integration of stochasticity, spatial transport and other mechanisms to reflect biological reality accurately.

Importantly, there are knowledge gaps about how the properties of dynamical systems map to specific developmental outcomes. Although multistability represents discrete cell fates, the precise trajectories and timescales by which cells transition between attractor states remain unresolved. Oscillation models elucidate molecular clock architectures, but how tissue-level oscillations robustly pattern embryonic fields lacks a unified mechanistic framework. Understanding how attractors and their manifolds are modulated by dynamic signals and morphogen gradients during inductive events is key to modelling cellular reprogramming comprehensively.

Looking ahead, dynamical systems theory will be pivotal for addressing frontiers, such as the encoding of positional information during body patterning, the developmental hourglass phenomenon of increasing and decreasing complexity, and principles of evolution's ability to flexibly tinker with embryonic regulatory architectures. Integrating dynamical modelling across multiple scales, from molecular networks to tissues and regenerating systems, holds transformative potential. Ultimately, realising this potential will hinge on cultivating interdisciplinary synergies between experimental and theoretical developmental biology.

## Supplementary Material

10.1242/develop.204617_sup1 Supplementary information

Interactive Python Code Python code used to generate the figures in the paper, including interactive simulations and parameter exploration analyses.
