# Supplementary material for: From genes to patterns: five key dynamical systems concepts to decode developmental regulatory mechanisms
Source: Development. 2025 Aug 1;152(14):dev204617. doi: 10.1242/dev.204617 (PMC12377817; doi:10.1242/dev.204617)
Supplement: Supplementary information [file develop-152-204617-s1.pdf]

Interactive Python Code

Python code used to generate the figures in the paper, including interactive simulations and parameter exploration analyses.

Available for download at
https://journals.biologists.com/dev/article-lookup/doi/10.1242/dev.204617#supplementary-data

Development • Supplementary information
