# Supplementary figures and images for: From genes to patterns: five key dynamical systems concepts to decode developmental regulatory mechanisms
Source: Development. 2025 Aug 1;152(14):dev204617. doi: 10.1242/dev.204617 (PMC12377817; doi:10.1242/dev.204617)

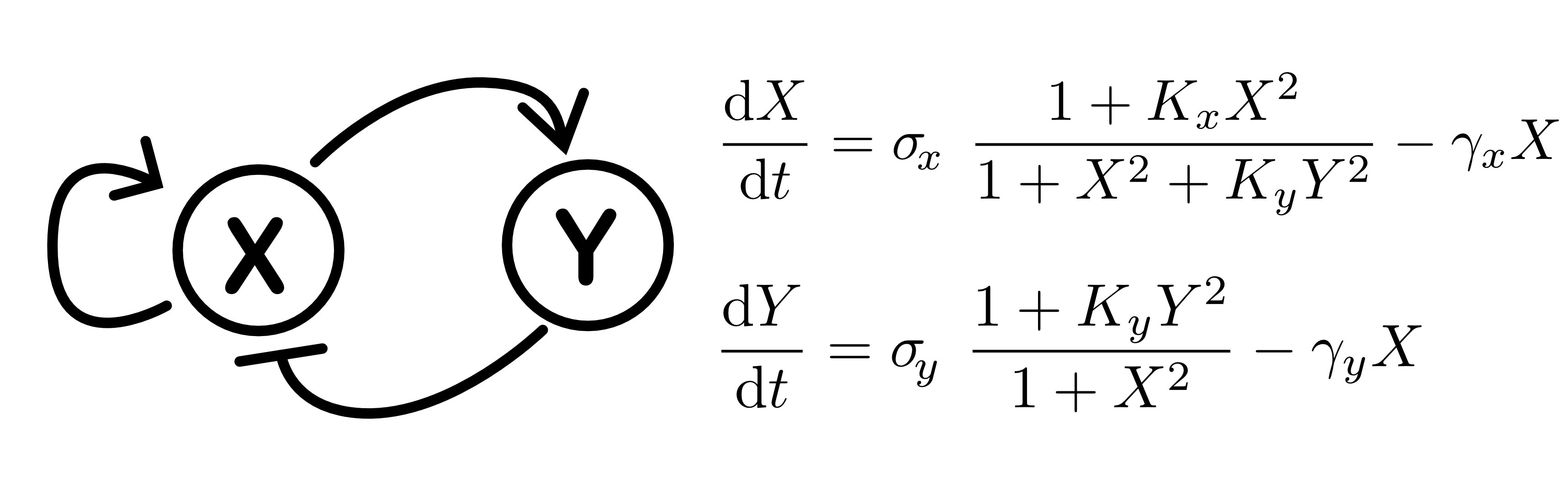

Supplement: Interactive Python Code [file develop-152-204617-Dataset1.zip › Supplementary_code/oscillating_network.jpg]

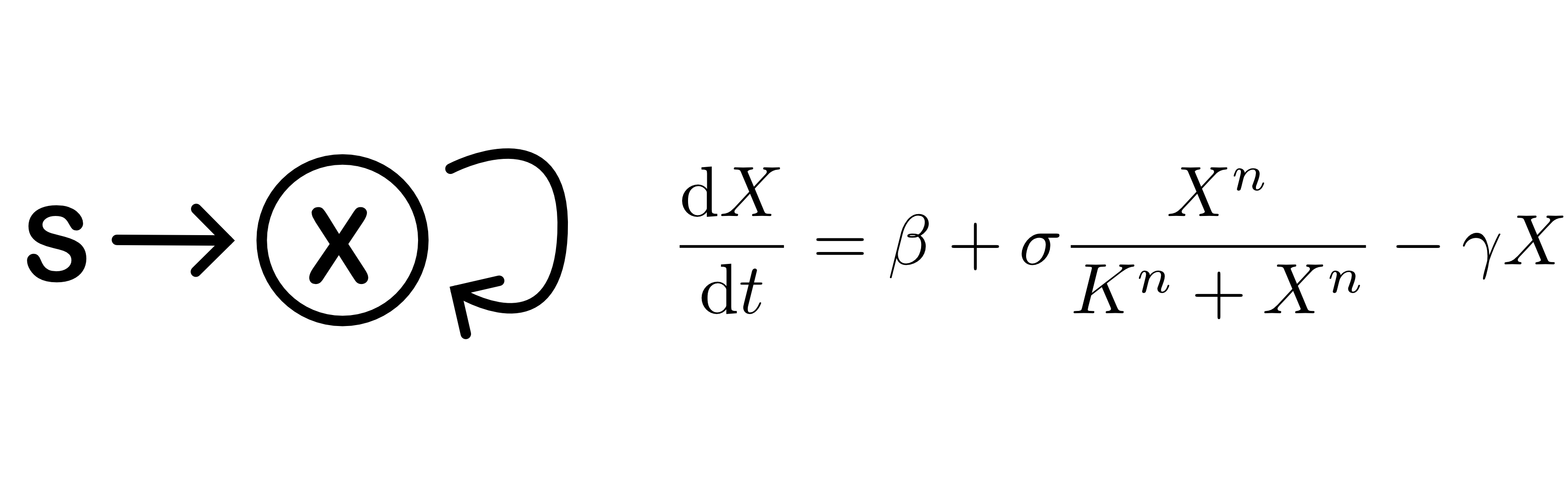

Supplement: Interactive Python Code [file develop-152-204617-Dataset1.zip › Supplementary_code/bistableswitch_network.jpg]
